# Fouling characterisation in PVDF membrane contactors for dissolved methane recovery from anaerobic effluents: effect of surface organofluorosilanisation

**DOI:** 10.1007/s11356-022-24019-z

**Published:** 2022-11-21

**Authors:** Ramón Jiménez-Robles, Vicente Martínez-Soria, Marta Izquierdo

**Affiliations:** Research Group in Environmental Engineering (GI2AM), Department of Chemical Engineering, School of Engineering, University of Valencia, Avda, Universitat S/N, 46100 Burjassot, Spain

**Keywords:** Fluoroalkylsilane, Hydrophobic membrane, Membrane fouling, Membrane stability, PVDF flat-sheet, Surface functionalisation

## Abstract

Characterisation of the fouling attached to PVDF membranes treating an anaerobic effluent for dissolved CH_4_ recovery was carried out. A commercial flat-sheet PVDF membrane and a PVDF functionalised by grafting of organofluorosilanes (mPVDF) that increased its hydrophobicity were subjected to a continuous flux of an anaerobic reactor effluent in long-term operation tests (> 800 h). The fouling cakes were studied by the membrane autopsy after these tests, combining a staining technique, FTIR, and FESEM-EDX, and the fouling extraction with water and NaOH solutions. Both organic and inorganic fouling were observed, and the main foulants were proteins, polysaccharides, and different calcium and phosphate salts. Also, a significant amount of live cells was detected on the fouling cake (especially on the non-modified PVDF). Although the fouling cake composition was quite heterogeneous, a stratification was observed, with the inorganic fouling mainly in the bulk centre of the cake and the organic fouling mainly located in the lower and upper surfaces of the cake. The mPVDF suffered a more severe fouling, likely owing to a stronger hydrophobic-hydrophobic interaction with the foulants. Irreversible fouling remained on both membranes after the extraction, although a higher irreversible fouling was detected in the mPVDF; however, a complete polysaccharide removal was observed. Regarding the operation performance, PVDF showed a lower stability and suffered a severe degradation, resulting in a lower thickness and perforations. Finally, the decrease in the methane recovery performance of both membranes was associated with the fouling depositions.

**Graphical Abstract:**

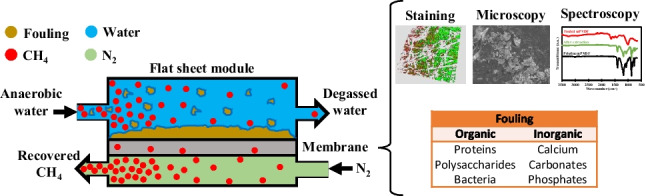

**Supplementary Information:**

The online version contains supplementary material available at 10.1007/s11356-022-24019-z.

## Introduction

Membrane contactor processes have shown great feasibility in different applications due to their high energy efficiency and small footprint (Jiménez-Robles et al. 2020; Lee et al. 2021; Liu et al. 2021b; Sohaib et al. 2022). Such applications include CO_2_ capture from biogas and flue gases, nutrient recovery from wastewaters, biobutanol recovery from fermentation broths, and CH_4_ degassing (Klaassen et al. 2005; Centeno-Mora et al. 2020; Davey et al. 2020; Zhang et al. 2020; Zhu et al. 2020). In an anaerobic digestion, more than 40% of the produced CH_4_ could be lost as dissolved CH_4_ (D-CH_4_) in the anaerobic effluent (AE) (Crone et al. 2016; Li et al. 2021; Stazi and Tomei 2021), leading to environmental and security issues (Lee et al. 2021; Stazi and Tomei 2021). In the last few years, membrane contactors for D-CH_4_ recovery from AE have been successfully implemented at the bench scale and at the prototype scale with a D-CH_4_ removal efficiency of up to 99% (Bandara et al. 2011; Cookney et al. 2016; Henares et al. 2016; Velasco et al. 2018; Rongwong et al. 2019; Sanchis-Perucho et al. 2020). The main challenge related to the implementation of this technology is the prevention of both fouling and wetting of the membranes in order to extend the membrane lifetime and maintain the performance. Such shortcomings could drastically increase the operational and maintenance costs (Wang et al. 2005; Rana and Matsuura 2010; Al-Juboori and Yusaf 2012; Al-Juboori et al. 2012; Rongwong et al. 2015; Yan et al. 2022). In this regard, the elucidation of fouling mechanisms in the membrane contactor has recently been identified as a critical issue that needs to be overcome for the application of this technology at the industrial level (Mansourizadeh et al. 2022).

The fouling phenomenon consists of the deposition or adsorption of unwanted compounds onto the membrane surface as well as in the membrane pores (Hu et al. 2014; Abdu et al. 2020; Lee et al. 2021). The different foulants can be classified (Chen et al. 2021; Costa et al. 2021; Liu et al. 2021b) into (i) inorganic matter and (ii) organic matter, which in biological processes are mainly related to growing microorganisms and the extracellular polymeric substances (EPSs) and soluble microbial products (SMPs) secreted by them, i.e. biofouling. The fouling layer on the membrane surface can induce an additional mass transfer resistance and/or a reduction in the useful lifetime of the membrane (Rana and Matsuura 2010; Lee et al. 2021; Liu et al. 2021b). The fouling grade relies on several factors related to membrane characteristics, hydrodynamic conditions, the nature of the treated water, the nature of the foulants, and the interaction forces between the membrane and the foulants (Rana and Matsuura 2010; Al-Juboori and Yusaf 2012; Yan et al. 2022). For hydrophobic membranes such as polyvinylidene fluoride (PVDF), polydimethylsiloxane (PDMS), and polypropylene (PP) used to treat different wastewaters and residual effluents, a strong interaction with organic matter is the main cause of fouling due to the hydrophobic nature of most organic compounds (Liu et al. 2021b). Therefore, proteins and polysaccharides are the major foulants (Rongwong et al. 2019; Chen et al. 2021). Furthermore, these organic foulants could hydrophilise the membrane surface, reducing the wetting resistance and promoting scaling and pore clogging (Zarebska et al. 2015; Liu et al. 2021b; Zhao et al. 2021).

The fouling may be reversible or irreversible depending on the type of interactions between the foulants and the membrane (Costa et al. 2021). In particular, microbial biofouling has been related to physically irreversible membrane fouling (Herzberg and Elimelech 2007; Chen et al. 2021). Different strategies have been proposed in order to prevent or minimise the negative effect of the fouling on the membrane performance, such as membrane cleaning procedures and disinfection of the feed (Al-Juboori and Yusaf 2012; Al-Juboori et al. 2012; Costa et al. 2021). Periodic cleaning with water could prevent or hinder the fouling development and irreversible attachment during long periods of operation (Henares et al. 2017). However, chemical cleaning with alkalis or acid solutions is usually needed to remove foulants strongly adhered to the membrane even though irreversible fouling is commonly reported after the cleaning procedures (Henares et al. 2017; Liu et al. 2021b). The amount of this irreversible fouling and the potential degradation of the membrane due to the attack of the foulants and chemical cleaners limit the useful lifetime of the membrane (Al-Juboori et al. 2012; Liu et al. 2021b; Pan et al. 2022).

To overcome the limitations related to fouling and wetting, new membrane synthesis and modification techniques have emerged with the goal of developing membranes with higher fouling and wetting resistance surfaces (Rana and Matsuura 2010). Thus, the fouling resistance can be improved by adding different additives in the synthesis step or by an ulterior modification of the membrane surface. In general terms, it is well accepted that the hydrophilisation of the membrane surface provides a higher fouling resistance for water treatment, avoiding the hydrophobic-hydrophobic interaction with organic foulants (Rana and Matsuura 2010; Lee et al. 2021). Thus, Sethunga et al. (2021) developed a membrane surface modification of PVDF, reducing its water contact angle (WCA) from 98.5 to as low as 14.0° and obtaining a surface with a higher fouling resistance for D-CH_4_ recovery from an AE. However, divergent results can be found in the literature because superhydrophobic membranes (WCA > 150°) that provide high fouling resistance in membrane distillation have more recently been reported (Liu et al. 2021b). In this regard, Abdu et al. (2020) synthesised a superhydrophobic PVDF (WCA of 164°) with a lower propensity to fouling compared with a conventional PVDF membrane with a lower hydrophobicity (WCA of 77°) when treating synthetic wastewater and actual seawater in membrane distillation at 60 °C.

In our recently published work (Jiménez-Robles et al. 2022b), we studied the performance of a modified PVDF membrane with enhanced hydrophobicity for D-CH_4_ recovery from an AE, and an improved performance and useful lifetime was inferred when comparing to the non-modified PVDF. Research works tackling the fouling effect on hydrophobic membranes treating AE for D-CH_4_ recovery are still very scarce. The works of Sanchis-Perucho et al. (2021) and Bandara et al. (2011, 2012) reported an insignificant fouling in a short-term period (90 min) with a PDMS membrane and in long-term experiments (> 30 days) with a polyethylene membrane, respectively. The negligible fouling reported in these works could be attributed to the dense structure of the membranes because a porous and rough membrane seems to be more susceptible to fouling deposition (Xu et al. 2019; Liu et al. 2021a; Zhao et al. 2021). Nevertheless, Henares et al. (2017, 2018) observed a similar decrease in the D-CH_4_ removal efficiency with microporous PP and dense PDMS membranes for a long-term period of > 200 h. Such divergent results could be explained by taking into account other operational factors such as the different hydrodynamic conditions of the fluid, since a higher turbulence hinders the fouling deposition onto the surfaces (Mikhaylin and Bazinet 2016). Furthermore, the fouling mechanisms in membrane contactor processes have been scarcely studied (Mansourizadeh et al. 2022). Rongwong et al. (2019) and Sethunga et al. (2021) characterised the fouling cake on modified PVDF membranes and reported that the compact fouling cakes were composed mainly of organic matter, with protein-like substances being the dominant foulants.

In this context, the main aim and contribution of this work was the determination of the fouling composition and potential mechanisms on PVDF membrane contactors with different hydrophobicity grades for the degassing of an anaerobic reactor effluent. For this purpose, fouling tests under a long-term operation were conducted with a flat-sheet membrane module using a commercial PVDF and an organofluorosilanisated PVDF with enhanced hydrophobicity. Then, the fouling cake was analysed in detail by means of different microscopy and spectroscopy techniques (FESEM-EDX and FTIR) in order to identify the different elements and compounds. Staining techniques were also used for the identification and quantification of proteins and polysaccharides and for the sensing of the metabolic status of the bacteria. In addition, a fouling extraction from the membrane was carried out with water and a NaOH solution in order to analyse the composition of the reversible and irreversible fouling. Finally, the influence of the fouling on the membrane performance and stability in D-CH_4_ recovery was also elucidated.

## Materials and methods

### Membrane material and functionalisation

The flat-sheet PVDF membrane was supplied by Dorsan Filtration S.L. (Spain). The membrane was composed of hydrophobic PVDF supported on a polyester (PET) non-woven support, resulting in a microporous structure with an overall porosity of 62 ± 3%, gravimetrically measured with 1-octanol (99%, Acros Organics, Germany) (Liu et al. 2016). The membrane pore size and liquid entry pressure were 0.2 µm and 1.8 bar, respectively, according to the supplier. The thickness and static WCA were measured, resulting in values of 159 ± 2 µm and 103.4 ± 1.6°, respectively.

The surface modification of the PVDF was carried out in a three-step procedure consisting of (1) activation with a NaOH solution, (2) functionalisation by means of a mixture containing 1H,1H,2H,2H-perfluorooctyltriethoxysilane (Dynasylan® F8261, Evonik GmbH, Germany) as the modifying agent and tetraethyl orthosilicate (TEOS, ≥ 99%, Sigma-Aldrich, USA) as the silica precursor, and (3) curing. A detailed description of the modification procedure can be found in our previous work (Jiménez-Robles et al. 2022b), and the modification conditions in each step were established and based on the optimal conditions that maximised the surface WCA, which were the following: activation with a 5% NaOH solution and functionalised with a concentration and ratio of Dynasylan/TEOS of 7.2% and 0.55, respectively. The modified PVDF (mPVDF) presented a thickness of 164 ± 1 µm and a WCA of 140.9 ± 2.5°. The overall porosity of the mPVDF was 59 ± 2%, similar to that of the non-modified PVDF.

### Experimental setup and operation procedure

Fouling tests using an AE as the liquid stream were conducted with both membranes for comparison purposes. The AE was collected from the anaerobic reactor of the urban wastewater treatment plant Quart-Benager II (Valencia, Spain), filtered with a 10–20 µm filter, and stored in the fridge (< 4 °C) before use in the tests. The characteristics of the filtered AE are provided in the Supplementary Material S1.

The fouling tests were carried out under long-term operation (> 800 h) using the laboratory-scale system depicted in Supplementary Material S2. Initially, a membrane sample was placed inside a circular flat-sheet module (FM) made of stainless steel with an effective contact area of 17.3 cm^2^ and a 2-L liquid feed tank was filled with AE. Then, a constant liquid flow rate (*Q*_*L*_) of 3.5 L h^−1^ was pumped in a closed loop through the liquid side of the FM using a peristaltic pump (Watson-Marlow Fluid Technology Solutions, UK) for up to around 830 h. *Q*_*L*_ was set based on our previous work (Jiménez-Robles et al. 2022a) and to promote the fouling deposition on the membrane because high fluxes increase the turbulence and favour the fouling mitigation (Mikhaylin and Bazinet 2016). At the end of the fouling tests, the membranes were extracted from the FM and subjected to different analysis techniques and methodologies (FESEM-EDX, FTIR, staining, and fouling extraction). In addition, water samples were taken at different times of operation to measure the concentration of proteins and polysaccharides and the chemical oxygen demand (COD).

### Methods for visualising and identifying the fouling on the membrane surface

#### FESEM-EDX

An inspection of the membrane surface and cross-section, as well as of the fouling cake deposited onto the membrane, was conducted by a field emission scanning electron microscope (FESEM) equipped with an energy dispersive X-ray (EDX) spectrometer with an accelerating voltage of 20 kV (Hitachi S4800, Hitachi Ltd., Japan). For the image acquisition, the membrane coupons were softly dehydrated in an ethanol series in a sequence of 50, 80, 100, and 100% for 3 min each and then air-dried overnight. Afterwards, the coupons were placed on an aluminium holder and then coated with a fine layer of Au/Pd by sputtering in a vacuum for 1 min. The FESEM-EDX technique was used to analyse the surface and cross-section morphology, thickness, and chemical composition of the membrane and the fouling cake.

#### FTIR

The surface chemical composition of the fouling cake was also studied by means of Fourier transform-infrared spectroscopy (FTIR) in the attenuated total reflectance mode (Cary 630 FTIR Spectrometer, Agilent Technologies, Inc., USA). The infrared spectra were recorded in the range of 4000–650 cm^−1^ and a resolution of 4 cm^−1^ and processed with the Agilent MicroLab FTIR software. For the infrared spectra acquisition, the membrane coupons were previously dehydrated with ethanol, as previously detailed.

#### Staining of proteins and polysaccharides

The identification and quantification of protein and polysaccharides attached to the membrane surface and fouling cake were carried out by means of staining and imaging with a confocal laser scanning microscope (CLSM) (Olympus FV1000, Olympus Corporation, Japan) equipped with a 10 × magnification objective. The SYPRO Orange Dye and the Concanavalin A conjugates to Alexa Fluor 633 (Molecular Probes, Inc., USA) were applied to stain proteins and polysaccharides, respectively.

The protein staining solution was prepared by diluting the SYPRO Orange Dye with 7.5%_v_ acetic acid (acetic acid 100%, VWR Chemicals, USA) until a volume ratio of 1:5000 was achieved. The polysaccharide staining solution was prepared at a concentration of 100 µg of Concanavalin A mL^−1^ using a phosphate-buffered saline solution (PBS) as the solvent.

For the staining technique, a 1 cm × 1 cm membrane coupon was first immersed in 1 mL of the protein staining solution and placed in a rotary shaker in the dark for 1 h at 75 rpm and room temperature. Then, the membrane was rinsed in 7.5%_v_ acetic acid to remove the excess staining solution (< 1 min) and then immersed in a PBS solution to remove the residual acetic acid (< 1 min). Afterwards, the membrane coupon was immersed in 1 mL of the polysaccharide staining solution and shaken for 1 h at 75 rpm at room temperature in the dark. Then, the membrane was rinsed in the PBS solution (< 1 min). Finally, the stained membrane coupon was air-dried with force aeration for ~ 2 h and stored in the dark at 4 °C.

The stained samples were observed with the CLSM, and at least two different series of images were taken at different depths with an imaged surface size of 1300 µm × 1300 µm. Each series of the CLSM images was processed with the *Imaris* software to reconstruct a 3D image (Herzberg and Elimelech 2007; Hu et al. 2014). In order to quantify the volume of the stained proteins and polysaccharides on the membrane, a 3D model was generated from the CLSM images also using the *Imaris* software (Hu et al. 2014). Thus, the specific volume of the foulant (proteins or polysaccharides) is defined as the total volume per unit of membrane area (µm^3^ µm^−2^) (Hu et al. 2014).

#### Water contact angle

The membrane surface hydrophobicity was evaluated by the static WCA. WCA measurements were carried out by the sessile drop technique (Hebbar et al. 2017). A water droplet of 5.5 ± 0.1 μL was deposited onto the membrane surface using a syringe pump (KF Technology s.r.l., Italy) at room temperature (~ 25 C). An image of the water drop profile was taken at 15 s with a digital microscope (Handheld Digital Microscope Pro, Celestron LLC, USA) under white light (Philips HUE Lamp, Koninklijke Philips NV, The Netherlands). ImageJ software was used for image processing using the *Contact Angle Plug-in* based on the ellipse approximation. The WCA was evaluated at different spots on the membrane, and a mean value was obtained from at least 10 measurements. Wet membranes were dried prior to the WCA measurements by removing the excess water and moisture with forced aeration at room temperature for approximately 2 h.

### Methods for the extraction and analysis of the membrane foulants in solution

#### Fouling extraction procedure

The fouling agents were also analysed after the detachment of the fouling cake from a membrane coupon of 8.7 cm^2^ using a sonication bath (Bransonic® 1510E-MT, Branson Ultrasonic Corporation, USA) with different solutions: a soft cleaning with 20-mL MilliQ water for 60 min and an alkaline cleaning with 20 mL of 0.01 M NaOH solution for 60 min. Then, the membrane was dried in an oven at 45 °C overnight. Both water and NaOH extraction solutions were analysed to measure the concentration of proteins, polysaccharides, phosphates, alkalinity, COD, total suspended solids (TSS), volatile suspended solids (VSS), and the metabolic status of the bacteria.

### Liquid sample analysis techniques

The characterisation of the filtered AE and the water and NaOH extraction solutions was conducted. The determination of TSS and VSS was carried out according to standard methods (Baird et al. 2018). Alkalinity was determined by means of the 5 pH point titration procedure (Moosbrugger et al. 1992) (848 Titrino Plus, Metrohm, Switzerland). The polysaccharide content was analysed by the phenol–sulfuric acid method proposed by Dubois et al. (1956) with glucose as the standard (Juang et al. 2010; Hafuka et al. 2019). The protein content was determined according to the modified Lowry methods by Onishi and Bar (Total protein kit, micro Lowry, Onishi & Bar modification, Sigma-Aldrich, USA) using bovine serum albumin as the standard. The COD and phosphate content were determined using colorimetric analysis kits (COD Cell Test 0–150 mg L^1^ and PO_4_^3−^ Cell Test 1.5–76.7 mg L^−1^, respectively, Merck Chemicals, Germany).

### Analysis of the bacteria viability

The metabolic status of the bacteria contained in the AE and in the fouling extraction solutions was evaluated to investigate the influence of the biofouling on the fouling cake development. The quantification of the live and dead cells was conducted by means of a flow cytometry using the LIVE/DEAD BacLight™ Bacterial Viability Kit L7012 (Molecular Probes Inc., USA) for the bacteria staining in the liquid samples. The viability kit is composed of the SYTO 9 and propidium iodide (PI) dyes to stain the live and dead cells, respectively. A staining solution was firstly prepared with a volume ratio of 1:1 (SYTO 9:PI) according to the specifications. Three µL of the staining solution was added to 1 mL of the liquid sample. The sample was vigorously shaken and incubated in the dark for 15 min at 200 rpm. Afterwards, the stained samples were filtered with a 50 µm filter and analysed in a flow cytometer (FACSVerse 3L 8C, Becton, Dickinson and Company, USA) equipped with a 488-nm laser and using BD Trucount™ Tubes (Becton, Dickinson and Company, USA) for the quantification of live and dead cells. Data acquisition and treatment were carried out with the FACSSuite software.

## Results

### Characterisation of the fouling deposited on the membrane surface

The evolution of the fouling formation on the membranes can be observed in the pictures taken from the PVDF and mPVDF membrane surfaces (Supplementary Material S3). The development of the fouling cake was clearly observed during the fouling tests. At the end of these tests, zones with a different fouling grade at the visual level on the membrane sample were selected for analysis.

### Staining of proteins and polysaccharides

**Fig. 1:**
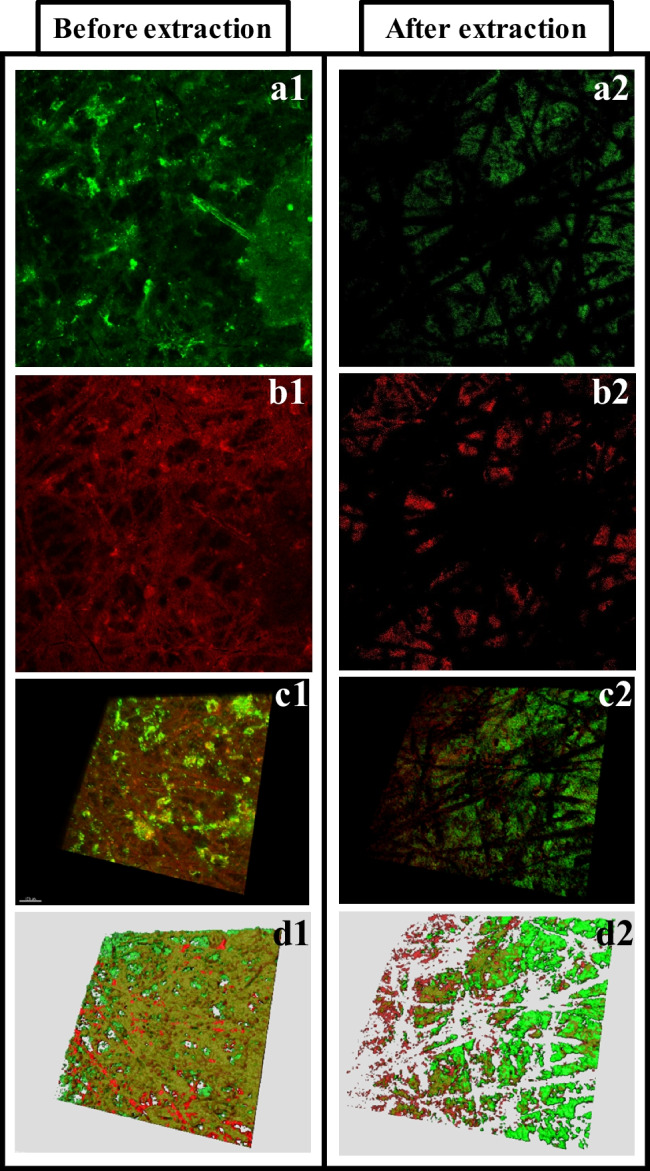
CLSM images of the staining of (**a**) proteins and (**b**) polysaccharides deposited on the non-modified PVDF membrane surface after the fouling test (before fouling extraction, **1**) and after the extraction (**2**). (**c**) Reconstruction in 3D and (**d**) 3D model of the protein and polysaccharide distribution. Proteins are in green and polysaccharides are in red. Imaged surface size of 1300 µm × 1300 µm

The distribution of the proteins and polysaccharides on the membranes was observed in the samples of PVDF and mPVDF at the end of the fouling tests. As an example, Fig. 1 shows a stained sample of the non-modified membrane (PVDF) before and after the fouling extraction in which the protein and polysaccharide distribution in 2-D (Fig. 1a and b), the 3-D image reconstruction (Fig. 1c), and the 3-D model (Fig. 1d) can be observed.

The CLSM images show the widespread presence of proteins and polysaccharides all over the fouling cake for both membranes before the extraction. After the fouling extraction, proteins and polysaccharides were also identified on the non-modified membrane (PVDF) (Fig. 1a2 and b2), but only proteins were found on the mPVDF membrane (images not shown). In both membranes, the distribution of proteins and polysaccharides on the surface was quite uneven with locations with a high concentration of them and others locations with hardly any of them (Fig. 1c1 and c2 for the non-modified PVDF).

The specific volumes of proteins and polysaccharides on the membrane surfaces before and after the fouling extraction obtained from the 3-D models are shown in Table 1 (examples of the models in Fig. 1d). These outcomes showed that the content of both foulants varied from different analysed surface locations (size of 1300 µm × 1300 µm) of the same membrane according to the heterogeneous distribution mentioned above. It is important to note that the polysaccharide content, unlike the protein content, was quite uniform along the surfaces in both membranes before the extraction. In this regard, surface locations of the membrane sample with a macroscopic darker brown colour presented a higher and lower protein content on the PVDF and mPVDF membranes, respectively, compared with those lighter brown zones before the extraction. After the fouling extraction, the protein content was always reduced and the complete removal of polysaccharides was observed in the mPVDF membrane. These outcomes could indicate a higher protein deposition rate on the membrane surface during the first steps of the fouling formation with a further coverage by other foulants, especially on the mPVDF membrane with a higher hydrophobicity.

**Table 1:** Specific volume ranges of proteins and polysaccharides (µm^3^ µm^−2^) on the non-modified (PVDF) and modified PVDF (mPVDF) membranes measured in different surface locations after the fouling test (time of operation > 800 h) with the anaerobic reactor effluent (before extraction) and after the fouling extraction. Imaged surface size of 1300 µm × 1300 µm

	Before extraction		After extraction	
	PVDF	mPVDF	PVDF	mPVDF
Proteins	27–37	21–29	10–12	12–15
Polysaccharides	15–17	18–19	9–21	n.d

n.d: not detected

### Fourier transform infrared spectroscopy (FTIR)

**Fig. 2:**
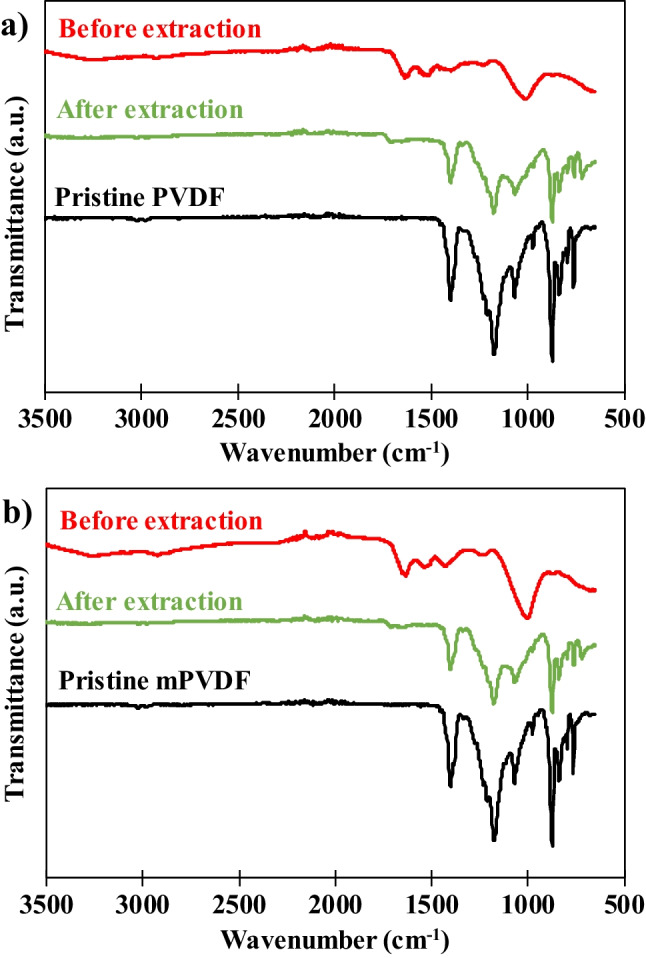
Fourier transform infrared spectra of the pristine, before fouling extraction (fouled), and after the fouling extraction surfaces of (**a)** PVDF and (**b)** modified PVDF (mPVDF) used in the fouling tests (operation time > 800 h). Transmittance is expressed in arbitrary units (a.u.)

The representative infrared spectra of the pristine PVDF and mPVDF membranes are shown and compared with the spectra after the fouling tests and after the fouling extraction in Fig. 2. The assignment of each band attributable to the foulants is shown in Table 2, and the representative bands of the spectra for the PVDF base material are shown in Supplementary Material S4. The spectra of both pristine PVDF and mPVDF membranes are very similar because the membrane modification occurred at mostly the surface level and FTIR-ATR analysis is not very surface sensitive (Rana and Matsuura 2010).

**Table 2:** Major FTIR absorption bands obtained in the analysis of both fouled PVDF and modified PVDF (mPVDF) membranes after the fouling tests (before the fouling extraction)

Wavenumber, cm^−1^	Band	Group	Reference
3250	Amide I (N–H stretching)	Proteins	(Dean 1998; Davis and Mauer 2010; Sairiam et al. 2013; Mohamed et al. 2017)
2922	C-H asymmetric stretching	Fatty acids	(Dean 1998; Davis and Mauer 2010; Mohamed et al. 2017)
1636	Amide I (C = O and C = N stretching)	Proteins	(D’Abzac et al. 2010; Mikhaylin and Bazinet 2016; Chen et al. 2018)
1533	Amide II (C-N stretching and N–H deformation)	Proteins	(Saha et al. 2007; D’Abzac et al. 2010; Zarebska et al. 2015)
1429	COO^−^ symmetrical stretching and phenolic C-O bond	Carboxylates and humic substances	(Zarebska et al. 2015; Chen et al. 2018; Yan et al. 2022)
1230	Phosphate ester asymmetric stretching (P = O)	Phospholipids and nucleic acids	(Davis and Mauer 2010; Xue et al. 2016)
1172	O–H stretching	Polysaccharides	(D’Abzac et al. 2010)
1010	C–O–C and C-O stretching	Polysaccharides	(Saha et al. 2007; Davis and Mauer 2010)
1000–800	Bands associated with polysaccharides and polysaccharide-like substances		(Gómez-Ordóñez and Rupérez 2011; Chen et al. 2018; Yan et al. 2022)

The spectra of the fouled membranes were significantly different with respect to the pristine state for both membranes, which can be attributed to the presence of a significant fouling cake on the membrane surface. In fact, most of the identified peaks in the fouled membranes can be related to organic compounds and/or microorganisms (biofouling) (Xue et al. 2016) according to the complex organic nature of the AE feed used in these experiments. Bands of functional groups attributed to proteins and polysaccharides were found at wavenumbers of 3250, 1700–1500, and 1200–800 cm^−1^. The peak at 2922 cm^−1^ was attributed to fatty acids involved in the metabolic activity of the bacteria, and the peak at 1429 cm^−1^ was attributed to the potential carboxylates generated in the anaerobic treatment, such as acetate, propionate, lactate, butyrate, and valerate (Cabrera-Rodríguez et al. 2017), and to humic substances. The presence of microorganisms such as bacteria was identified by the band at 1230 cm^−1^ that represents phospholipids and nucleic acids.

Similar infrared spectra of both membranes were obtained even though higher band intensities were usually observed for the mPVDF membrane, suggesting a higher foulant deposition as visually identified (Supplementary Material S3). It could be noted that the intensity of the band at 1230 cm^−1^ was quite similar, which could denote a lower proportion of microorganisms in the fouling cake of the mPVDF membrane.

After the fouling extraction, the obtained infrared spectra were similar to those of the pristine state for both membranes (Fig. 2). Bands owing to the PVDF base material presented lower intensities in both spectra after the fouling extraction, and a new peak appeared at ~ 700 cm^−1^, which could be related to EPSs (Chen et al. 2018). These outcomes indicated that an irreversible fouling covered the membrane surface composed of protein- and/or polysaccharide-like compounds, in accordance with the light brown colour observed on the membrane after the extraction (Supplementary Material S3). Comparing both membranes, mPVDF presented zones with a higher irreversible fouling grade at the visual level because a darker brown colour was observed, and the infrared spectra in these zones showed the characteristic band for proteins at ~ 1600 cm^−1^. This could indicate that antifouling properties dropped upon making the surface more hydrophobic. Additionally, a new band appeared at 1707 cm^−1^ after the extraction, which could be attributed mainly to the carbonyl group (C = O) from the PET support of the membranes, suggesting a surface degradation, especially in the PVDF membrane.

### Microscopy and X-ray spectroscopy (FESEM-EDX)

**Fig. 3:**
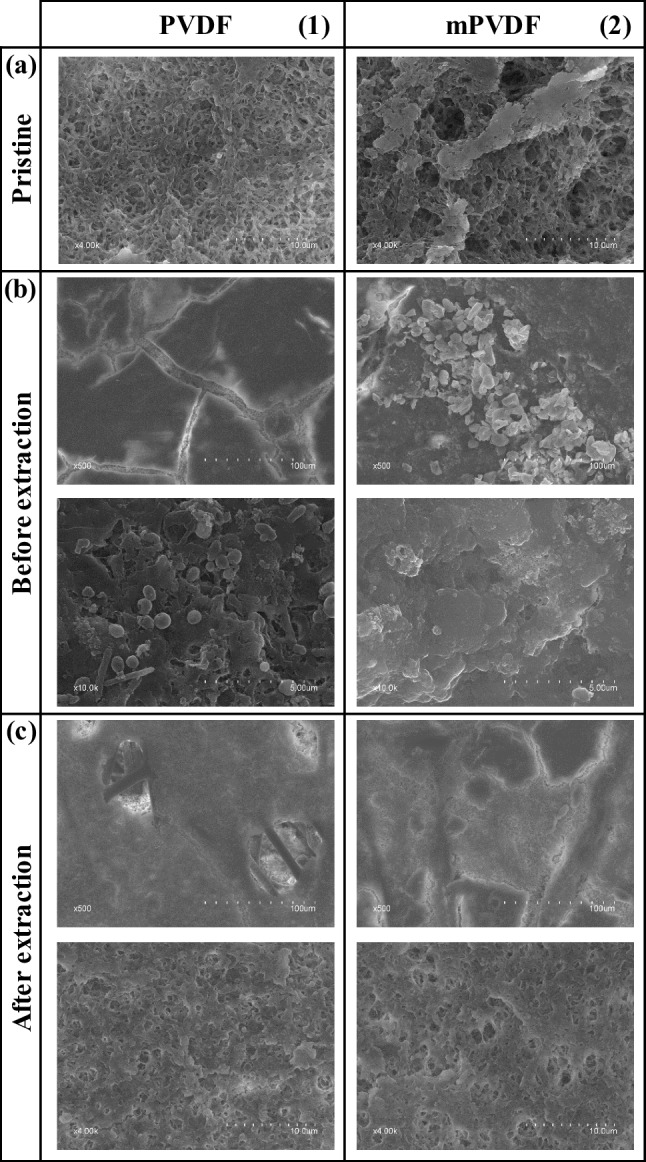
FESEM images of the surface of the (**1**) PVDF and (**2**) modified PVDF (mPVDF) captured from the (**a**) pristine membranes, (**b**) fouled membranes before the fouling extraction (operation time > 800 h), and (**c**) after the extraction

The morphology of the surface and cross section of the PVDF and mPVDF membranes was analysed by FESEM, and the chemical composition was also determined by EDX. The surface morphology of the pristine membranes and the membranes after the fouling tests and the fouling extraction is shown in Fig. 3. The membranes were clearly covered by a dense-like fouling cake, leading to a severe reduction in the surface porosity (Fig. 3a and b). Some cracks on the fouling cake can be visualised due to the drying process (Fig. 3b1). The presence of spheres and needle-like biomass particles was quite noticeable on the PVDF membrane (Fig. 3b1) and could be associated with *Bacillus and Streptococcus*, respectively. On the contrary, a higher content of larger crystals was observed on the mPVDF membrane than on the PVDF membrane (Fig. 3b2).

After the fouling extraction, perforations were observed all over the PVDF membrane surface (Fig. 3c1), indicating a severe degradation of the top layer, i.e. the PVDF material, because the PET non-woven support was clearly identified through these perforations. The rest of the surface suffered a decrease in the surface porosity owing mainly to the irreversible fouling mentioned above (Fig. 3a1 and c1). The mPVDF membrane also experienced a similar decrease in surface porosity (Fig. 3a2 and c2) even though the severe degradation and perforations on the top layer were not detected as in the PVDF membrane (Fig. 3c).

**Fig. 4:**
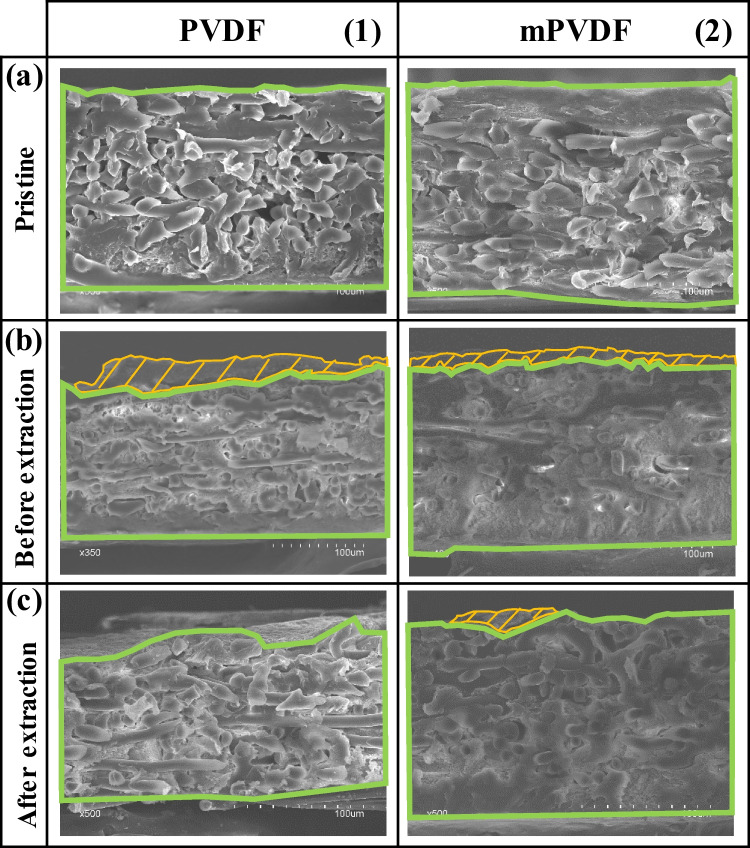
FESEM images of the cross section of the (**1**) PVDF and (**2**) modified PVDF (mPVDF) captured from the (**a**) pristine membranes, (**b**) fouled membranes before the fouling extraction (operation time > 800 h), and (**c**) after the extraction. The boundaries of the membrane and fouling cake are highlighted in green and orange, respectively

The FESEM images of the cross section are shown in Fig. 4. The bulk of the fouling cake observed on the membranes was dense-like, and the measured overall membrane thickness was somewhat uneven, especially on the PVDF membrane, as reported by other authors (Juang et al. 2010). Compared to the pristine PVDF (159 ± 2 µm), the non-uniform deposition of foulants resulted in thicker sections with values up to 195 µm due to the fouling cake (Fig. 4b1) and thinner sections with values as low as 134 µm. These low thickness values suggested the degradation of the membrane during the long-term operation. The reduction in the membrane thickness was confirmed from the FESEM images of PVDF after the fouling extraction because a quite irregular surface was observed with an overall thickness of 142 ± 16 µm (Fig. 4c1). It should be noted that in Fig. 4c1, the membrane surface can be seen as background above the upper cross-section boundary due to the membrane degradation. In the case of mPVDF (Fig. 4b2), the fouling cake appeared more uniformly distributed with a maximum overall membrane thickness of up to 187 µm. After the fouling extraction (Fig. 4c2), a thickness of 161 ± 2 µm was measured, similar to the pristine mPVDF (164 ± 1 µm).

The surface chemical composition of the pristine membranes and the membranes after the fouling tests and the fouling extraction are shown in Table 3. The surface F content drastically decreased in both membranes after the fouling tests, which is attributed to the formation of a fouling cake that masks the top membrane surface, as observed by other authors (Yan et al. 2022). Comparing the fouled membranes, a lower F content on mPVDF with respect to the PVDF was observed, which indicates a higher coverage grade with fouling on the mPVDF. The O content increased to values of around 26% and 37% in PVDF and mPVDF, respectively, and an N content of around 5% was observed, which could be related to the presence of biomass, protein, and protein-like substances (Al-Juboori and Yusaf 2012; Rongwong et al. 2019). In addition, the predominant content of O and C denoted mainly an organic fouling and high O to C ratios (O/C) suggested the presence of EPSs in both membranes (Khan et al. 2010; Zarebska et al. 2015). This high content of O and C could also indicate a scaling of carbonate and hydroxide salts. Ca, P, and Mg represented the other major elements found on the surface, and a higher content of these elements was observed on the mPVDF. The Ca was found all over the surface likely in the form of carbonates, and also particles composed mainly of P and Mg were detected, which could be related to crystals of struvite ((NH_4_)MgPO_4_) or struvite analogues (P-Mg) (Choo and Lee 1996). In addition, other minor elements and particles of aluminosilicates and others composed of Cu–Zn-Na were detected on the surface. FESEM-EDX images with the distribution of the different elements and composition of particles on the membrane surfaces can be found in Supplementary Material S5.

**Table 3:** EDX analysis of the membrane surface and the fouling cake cross section from the fouled PVDF and modified PVDF (mPVDF) after the fouling test (> 800 h) and after the fouling extraction. Values expressed in %_wt_. The errors denote the standard deviation of at least 3 measurements in different spots

Element	PVDF				mPVDF			
	Membrane surface			Fouling cake cross section	Membrane surface			Fouling cake cross section
	Pristine	Before extraction	After extraction	Before extraction	Pristine	Before extraction	After extraction	Before extraction
F	52.67 ± 5.90	17.24 ± 6.40	51.06 ± 0.41	0.00 ± 0.00	54.94 ± 6.50	6.45 ± 1.76	46.39 ± 4.31	1.36 ± 1.94
C	43.32 ± 5.00	39.23 ± 1.84	39.67 ± 1.11	6.57 ± 6.89	41.16 ± 5.40	29.91 ± 1.24	34.86 ± 1.24	21.63 ± 9.55
O	3.61 ± 0.60	26.37 ± 3.17	6.92 ± 0.50	4.16 ± 6.02	3.45 ± 0.70	36.52 ± 1.85	11.63 ± 2.71	8.67 ± 2.45
N	-	5.97 ± 0.92	0.00 ± 0.00	0.00 ± 0.00	-	4.90 ± 0.06	0.74 ± 0.23	0.56 ± 0.57
Ca	-	5.79 ± 0.35	0.84 ± 0.25	67.20 ± 12.55	-	9.66 ± 3.32	3.03 ± 1.35	43.92 ± 9.81
P	-	3.15 ± 0.26	0.45 ± 0.14	10.46 ± 2.24	-	6.21 ± 0.37	1.58 ± 0.69	15.78 ± 2.68
Mg	-	0.78 ± 0.26	0.22 ± 0.03	0.00 ± 0.00	-	2.54 ± 1.27	0.21 ± 0.04	0.21 ± 0.12
Si	-	0.12 ± 0.02	0.08 ± 0.02	0.73 ± 1.26	0.46 ± 0.10	0.21 ± 0.05	0.29 ± 0.02	0.01 ± 0.01
Na	-	0.31 ± 0.04	0.46 ± 0.02	0.00 ± 0.00	-	0.95 ± 0.34	0.34 ± 0.09	0.00 ± 0.00
K	-	0.04 ± 0.04	0.01 ± 0.01	0.26 ± 0.31	-	0.22 ± 0.09	0.01 ± 0.01	0.07 ± 0.04
Fe	-	0.28 ± 0.03	0.07 ± 0.02	4.18 ± 1.74	-	0.41 ± 0.07	0.10 ± 0.05	1.61 ± 0.46
Cu	-	0.18 ± 0.03	0.12 ± 0.02	1.74 ± 0.85	-	0.27 ± 0.05	0.22 ± 0.09	0.79 ± 0.23
Zn	-	0.00 ± 0.00	0.00 ± 0.00	3.49 ± 1.06	-	0.47 ± 0.15	0.47 ± 0.21	2.62 ± 0.62
Al	-	0.15 ± 0.06	0.05 ± 0.01	0.00 ± 0.00	-	0.12 ± 0.03	0.04 ± 0.02	1.40 ± 0.64
S	-	0.39 ± 0.06	0.06 ± 0.02	0.65 ± 0.11	-	0.46 ± 0.03	0.10 ± 0.05	1.35 ± 0.47
Cl	-	0.00 ± 0.00	0.00 ± 0.00	0.54 ± 0.93	-	0.70 ± 0.40	0.00 ± 0.00	0.00 ± 0.00

After the fouling extraction, a surface F content lower than that of the pristine membranes was recorded from both membranes. This decrease in the F content could be attributed to the presence of the irreversible fouling and/or to a loss of F atoms due to the membrane degradation by the foulants or to the dragging effect by the liquid flux stress (Jiménez-Robles et al. 2022b). The membrane surface degradation in the PVDF was also identified from the absence of F inside the perforations that appeared after the fouling removal mentioned above (distribution of F atoms shown in Supplementary Material S6). The F content detected on the surface was lower for the mPVDF, likely owing to a higher amount of irreversible foulants that masked the membrane surface. Ca, P, Mg, and N remained as the main elements of the irreversible fouling even though N was not found on PVDF. Moreover, the ratio O/C remained higher than that in the pristine membranes, which could indicate the presence of EPS. Other minor inorganic elements were not completely removed after the extraction, which could suggest the need for an additional acid-cleaning step.

The cross section of the fouling cakes deposited on both membranes was also analysed, and the results are shown in Table 3. FESEM-EDX images of the distribution of F, Ca, and P are shown in Supplementary Material S7 for both membranes. The fouling cake cross section was composed mainly of Ca, P, and C. Comparing the results from the surface and cross section, a significantly higher inorganic fouling was detected in the centre bulk of the cake, denoting a stratification of foulants across this cake. The low N content with values ≤ 1% and the higher amount of Ca in the cross section of both membranes could indicate a Ca scaling that was furthered covered by a microbial and organic layer, as reported by other authors (Chen et al. 2021). Other minor elements presented a higher content with respect to the surface, such as Fe, Cu, and Zn, promoting an inorganic type of fouling.

### Water contact angle analysis

**Fig. 5:**
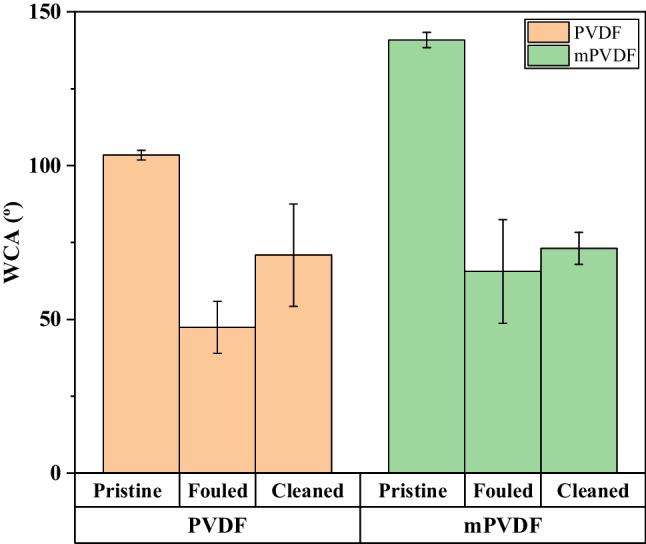
Variation in the static water contact angle of the pristine, fouled (before the fouling extraction), and cleaned (after the fouling extraction) PVDF and modified PVDF membranes (mPVDF)

The surface hydrophobicity was evaluated by measuring the static WCA of both membranes after the fouling tests and after the fouling extraction, and the results are presented in Fig. 5. A lower WCA was observed after the fouling tests, with a decrease of more than 50% from the initial value for both membranes, and the WCA measurements showed a large deviation, owing mainly to the heterogeneous formation of the fouling cake. This decrease in the WCA can be attributed to the hydrophilisation promoted by some fouling agents (Zarebska et al. 2015), masking the membrane surface properties (Khan et al. 2010; Zhao et al. 2021). After the fouling extraction, the WCA increased to 70.9 ± 16.6° and 73.1 ± 5.2° for the PVDF and mPVDF, respectively, representing 69% and 55% of their initial WCA. Thus, an incomplete extraction of the foulants or membrane degradation can be inferred, as the initial hydrophobicity grade was not restored. It is noteworthy to highlight that the increase in the WCA after the fouling extraction of the mPVDF was almost negligible, which suggests the presence of a high irreversible fouling grade and/or a stronger foulant-membrane interaction.

### Fouling analysis in the extraction solution

The solutions obtained from the extraction of the fouling cake with MilliQ water and a 0.01 M NaOH solution were analysed, and the results are shown in Table 4. The presence of both organic and inorganic matter as well as microorganisms was detected. The soluble COD and the presence of proteins, polysaccharides, and VSS were indicators of organic fouling, and the presence of microorganisms was confirmed by the significant amount of live and dead cells detected in all the extraction solutions. Most of the foulants were detached in the first sonication with water, though the second extraction with NaOH also contributed to the membrane cleaning, especially removing organic foulants. The extracted inorganic fouling was mainly recovered with water, as indicated by the higher alkalinity and PO_4_^3−^ concentration in the water extraction solutions (around 180 µg CaCO_3_ cm^−2^ and 100–170 µg PO_4_^3−^ cm^−2^), which indicated the presence of carbonate, bicarbonate, and phosphate salts. A significantly higher content of phosphates was extracted from the fouling cake of PVDF with respect to the mPVDF, and also a higher content of live and dead cells was detected, which could indicate the presence of a greater biomass. The extracted TSS from the PVDF membrane was associated with the organic matter (where TSS = VSS), so the total extracted inorganic foulants from the PVDF were mainly dissolved. In the case of mPVDF, 25% of the TSS extracted corresponded with the inorganic solids.

**Table 4:** Characterisation of the foulants in the extraction solutions (MilliQ water and 0.01 M NaOH solution) from the PVDF and modified PVDF membrane (mPVDF). Results are expressed per unit of membrane area

	PVDF			mPVDF		
	Water	NaOH	Total	Water	NaOH	Total
COD*, µg O_2_ cm^−2^	109.8	0.0	109.8	98.3	28.9	127.2
Alkalinity*, µg CaCO_3_ cm^−2^	147.1	36.8	183.9	144.9	38.6	183.5
Proteins*, µg cm^−2^	45.3	13.4	58.7	48.7	6.7	55.4
Polysaccharides*, µg cm^−2^	31.1	5.3	36.4	38.2	7.1	45.2
PO_4_^3−*^, µg cm^−2^	164.7	2.9	167.6	101.2	0.0	101.2
TSS, µg cm^−2^	0.18	0.00	0.18	0.72	0.05	0.77
VSS, µg cm^−2^	0.18	0.00	0.18	0.53	0.05	0.58
Number of live cells*, cells cm^−2^ 10^−5^	38	4	41	10	6	16
Number of dead cells*, cells cm^−2^ 10^−5^	22	1	23	8	1	9

*Measured after sample filtration (0.7 µm)

## Discussion

### Characterisation of the fouling deposited on the membranes

Both PVDF and mPVDF membranes were susceptible to fouling when treating an AE for D-CH_4_ recovery. Visually, the fouling content increased with the time of operation owing to a continuous deposition of foulants from the anaerobic water and/or biofilm growth, as also reported by other authors (Hu et al. 2014). The decrease in the WCA at the end of the fouling test was in agreement with the fouling cake development in which WCA decreased as the fouling increased. This surface hydrophilisation can be especially favoured by the deposition of amphiphilic proteins and polysaccharides with hydrophilic moieties such as O–H, N–H, and COO, which are susceptible to the formation of hydrogen bonds with water molecules (Saha et al. 2007; Zarebska et al. 2015; Chen et al. 2021; Costa et al. 2021). Also, the biofilms, metal oxides, and alumminosilicates contribute to the hydrophilisation, inducing wetting (Costa et al. 2021; Zhao et al. 2021). In addition, the potential loss of F atoms from the membrane surface owing to the degradation observed in the FESEM images was especially severe on the PVDF, contributing to the decrease in the WCA.

Both organic and inorganic fouling were found on the membrane, and proteins and polysaccharides were the major organic foulants. The presence of proteins in the fouling cake was in accordance with the decrease observed in the protein content in the analysed anaerobic water samples from the fouling tests collected at different times of operation. The decrease in the protein content was more pronounced in the anaerobic water used for the PVDF, with a decrease of 12% in the first 3 days, whilst a decrease of less than 8% was observed in the test with the mPVDF. This result was in accordance with the higher protein content detected on PVDF at the end of the fouling test. By contrast, the variation in the polysaccharide content in the anaerobic water was not significant. Also, a slight decrease (5–10%) in the COD of the anaerobic water used in the fouling tests with the time of operation was observed in both membranes, which also suggested a deposition of organic matter on the membranes. Henares et al. (2017, 2018) also analysed the anaerobic water stream at the inlet and outlet of different hollow fibre membrane contactors and reported a decrease in the VSS, proteins, and turbidity at the outlet of the contactor attributed to the fouling deposition. Also, other authors have qualitatively identified the aromatic proteins and SMP substances as the main organic matter in the fouling extraction solution when treating AEs (Rongwong et al. 2019; Sethunga et al. 2021).

The fouling could be aggravated by the microbial biofouling because the large number of live cells detected in the extraction solution could also promote the generation of EPSs and other SMPs. Thus, the higher protein content observed on the fouling cake surface of the PVDF could also be attributed to the presence of a higher number of live cells. The grafting of fluoroalkyl chains on the PVDF could be responsible for the lower number of live and dead cells determined in the mPVDF because the microbes present a much weaker interaction with this chemical structure, which reduces the biofouling (Zhu et al. 2020).

The inorganic fouling was composed mainly of Ca, P, and Mg. Other authors have also reported those elements as the major inorganic foulants from different wastewaters in membrane distillation (Yan et al. 2022). Other minor elements found, such as Na, Cu, Zn, Fe, Al, Si, and S, could involve different types of scaling, as observed by other authors (Zarebska et al. 2015; Chen et al. 2021). In addition to the inorganic particles detected in the fouling cake, other salts could be deposited, such as metal hydroxides and oxides (Mikhaylin and Bazinet 2016; Zhao et al. 2021). The differences between the surface and centre bulk composition of the fouling cake denote a clear stratification of foulants across the cake with predominantly inorganic fouling in the bulk centre and organic fouling mainly located on the surfaces of the cake. Therefore, the fouling cake showed a complex and non-uniform matrix of several inorganic and organic/biological foulants that could hinder the cleaning procedures needed to maintain the membrane performance and avoid early membrane deterioration.

The surface morphology of the dense-like fouling cake was similar to those reported by other authors on distillation membranes (Chen et al. 2021; Pan et al. 2022; Yan et al. 2022) and on reverse osmosis membranes (Herzberg and Elimelech 2007; Zhao et al. 2021) treating different types of wastewaters. In these works, organic matter deposition and adsorption of EPS secreted by microorganisms have been reported as well as a decrease in the membrane surface porosity.

Regarding the fouling removal from the membrane surfaces, MilliQ water was firstly used in order to simulate a soft and desirable cleaning and to avoid any interaction and deterioration of the membrane owing to the cleaning agent attacks (Henares et al. 2017, 2018). A second alkaline sonication was applied because a residual fouling cake with a brown colour was observed on the membrane surface that was mainly attributed to organic and biofilm fouling. This second sonication with a NaOH solution was conducted because an alkali solution is usually proposed to remove organic fouling such as polysaccharides, proteins, peptides, fatty acids, humate, and surfactants (Mikhaylin and Bazinet 2016; Henares et al. 2018; Hafuka et al. 2019; Costa et al. 2021). Finally, the irreversible fouling observed on the membranes was mainly attributed to a strong hydrophobic-hydrophobic interaction of the proteins and polysaccharides with the membrane (Hu et al. 2014), especially with the mPVDF with a higher hydrophobicity. Additionally, the outcomes from the EDX analysis suggest the need for an additional cleaning step to enhance the removal of inorganic compounds so that a cleaning with weak organic acid solutions could be proposed (Mikhaylin and Bazinet 2016; Henares et al. 2017, 2018).

Especially in the case of PVDF, the irreversible fouling could also be favoured by the penetration of the foulants in the pores (Juang et al. 2010; Hu et al. 2014) because the membranes with a lower WCA and wetting resistance are more susceptible to pore blocking (Abdu et al. 2020). Simultaneously, fouling could also aggravate the wetting phenomenon (Zarebska et al. 2015; Chen et al. 2021) due to hydrophilic moieties of some other additional foulants such as fatty acids or surfactants (Li et al. 2021). Also, the organic compounds in the treated anaerobic water may reduce the surface tension of the liquid, leading to an increase in the membrane wetting (Henares et al. 2018). Therefore, cleaning strategies should be evaluated in future studies with a special focus on the prevention of irreversible fouling and improvement in the membrane lifetime.

The prior organofluorosilanisation conducted over the PVDF led to a membrane with a greater stability under long-term operation because no significant degradation and a higher WCA were observed on mPVDF after the fouling tests in spite of the higher fouling compared to PVDF. By contrast, the PVDF membrane even experienced a water breakthrough at ~ 800 h, resulting in a shorter useful lifetime. That suggests that the functionalisation layer protected the surface against the potential damage from foulants and/or cleaning agents. Nevertheless, the greater hydrophobicity of the mPVDF seemed to promote the fouling development and hamper the detachment of the foulants, attributed mainly to the stronger hydrophobic-hydrophobic interaction between hydrophobic moieties of the proteins and polysaccharides and the membrane surface (Rongwong et al. 2019). That was corroborated by several findings after the fouling extraction: (i) the darker colour of the irreversible fouling cake on the mPVDF suggesting a higher amount of foulants, (ii) higher content of the foulants analysed by the EDX than for the PVDF, (iii) a higher organic matter content in the fouling extraction solution from mPVDF, and (iv) a negligible restoration of the WCA. Nevertheless and according to the staining analysis, the absence of polysaccharides in the irreversible fouling cake on the mPVDF suggests lower adhesion forces between polysaccharides and the modified surface and/or an improvement in the cleaning/removal efficiency of this type of foulant from organofluorosilanisated PVDF. Thereby, polysaccharide fouling was totally reversible when using the mPVDF.

In general terms, the deposition tendency of the foulants and, therefore, the fouling cake development on both PVDF and mPVDF seemed similar because similar results were obtained. Thus, these outcomes persuaded to conclude that protein and polysaccharide deposition was more dominant in the first stage of the fouling formation according to the large hydrophobic interaction forces with the membrane surface. Then, this deposition led to a conditioning layer and promoted the adherence of other foulants, including inorganic compounds and microorganisms, as reported by other authors (Al-Juboori and Yusaf 2012; Hu et al. 2014). Therefore, this initial deposition could explain the high amount of inorganic elements such as Ca and P located in the bulk of the fouling cake even though the organic matter dominates the most external parts of the layer, as indicated by the EDX analysis.

### Effect of the fouling on the membrane performance for dissolved methane recovery

**Fig. 6:**
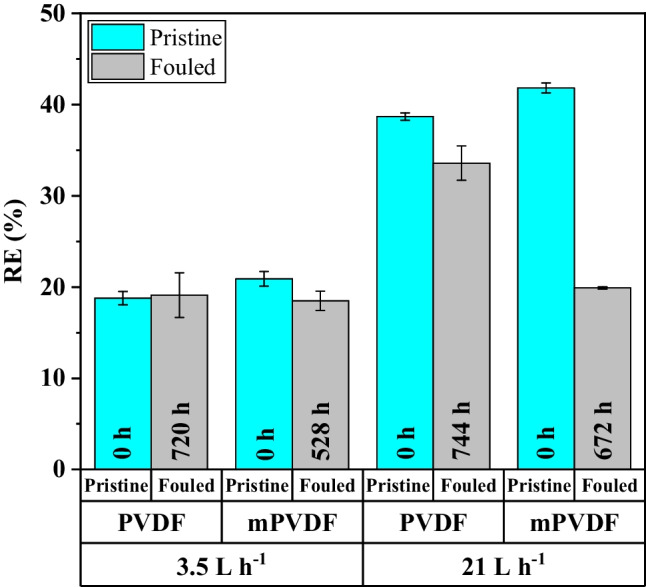
Effect of the fouling time on the dissolved CH_4_ removal efficiency (RE) in degassing tests with the PVDF and modified PVDF (mPVDF) at different liquid flow rates. Data from Jiménez-Robles et al. (2022b)

In order to elucidate the effect of the fouling cake on the membrane performance, degassing tests for D-CH_4_ recovery with the pristine and fouled PVDF and mPVDF membranes at different times of operation and liquid flow rates during the fouling tests using the same AE feed were studied in our previous work (Jiménez-Robles et al. 2022b). The removal efficiency (RE), which is defined as the percentage of recovered D-CH_4_ from the liquid feed respect to the initial D-CH_4_, was significantly affected by the presence of a fouling cake at the highest liquid flow rate tested of 21 L h^−1^ (Fig. 6). The mPVDF with a higher fouling experienced a 50% decrease in the RE after 672 h of operation, whilst the RE only decreased 15% with the PVDF after 744 h. On the other hand, at the lowest liquid flow rate of 3.5 L h^−1^ the RE kept almost constant even after the fouling cake formation, mainly attributed to the limiting mass transfer resistance located in the liquid phase (Li et al. 2021; Sanchis-Perucho et al. 2021; Velasco et al. 2021; Jiménez-Robles et al. 2022a). The RE decline observed during the long-term operation indicated an additional mass transfer resistance provided by the fouling cake (Henares et al. 2017). The wetting phenomenon induced by some foulants should also be taken into account in the increase of the overall mass transfer resistance, as mentioned above.

From the outcomes presented, a proper cleaning strategy of membranes is required to preserve the membrane performance. For the cleaning protocols, it is essential to consider the nature and concentration of foulants in the feed when choosing the cleaning agents in order to optimise the frequency and chemical concentration (Costa et al. 2021). Therefore, membrane structural damages and exacerbated maintenance costs could be avoided. Taking into account the fouling cake development on hydrophobic PVDF membrane contactors treating AE elucidated in this work, the following preventive cleaning protocol could be tested in future studies: daily flushing with a diluted alkaline cleaning solution for 5–10 min followed by flushing with pure water for 5–10 min and a weekly flushing with ≤ 1% citric acid (Henares et al. 2018) and biocide solutions (Mikhaylin and Bazinet 2016) for 5–10 min. All cleaning solutions should be applied with a flow rate representing the 90–95% capacity of the membrane contactor in order to increase the turbulence. However, further studies are required to evaluate and optimise a suitable cleaning protocol for each membrane and operational condition.

## Conclusions

A commercial PVDF membrane and an organofluorisilanisated PVDF membrane with enhanced hydrophobicity experienced a fouling cake formation on their surfaces when treating an anaerobic reactor effluent for dissolved methane recovery during a long-term operation (> 800 h). Organic and inorganic fouling were identified in a somewhat heterogeneous fouling cake. Proteins and polysaccharides were the major organic foulants and were mainly located at the surface of the membrane and of the fouling cake. Inorganic fouling was composed mainly of salts of carbonate, calcium, and phosphate and was especially located in the centre bulk of the fouling cake, which indicates a stratification of the foulants.

The modified PVDF suffered a higher fouling deposition due to a stronger foulant-membrane interaction. However, the polysaccharides were completely removed from the mPVDF, a lower fouling related to biomass was inferred, and a higher membrane stability was demonstrated. Therefore, the functionalised surface layer protected the membrane from the potential foulants and/or damaging attack of the cleaning agents.

In conclusion, hydrophobic organic matter was initially attached to the hydrophobic membrane surface with a subsequent adhesion of microorganisms and especially a high deposition of other inorganic foulants. A preventive cleaning protocol should be established to avoid the loss of membrane performance during a long-term operation.

Regarding the membrane performance, the decline in methane recovery during the long-term operation was also related to the fouling cake formation, and consequently, to the additional resistance for the mass transport. Thus, the decline in methane recovery was more pronounce with the mPVDF, presenting a lower fouling resistance.

## Supplementary Information

Below is the link to the electronic supplementary material.

Supplementary file1 (DOCX 4922 KB)

## Data Availability

The authors declare that the relevant data supporting the findings of this study are available within the article and its supplementary information files.
