# Supplementary material for: Fouling characterisation in PVDF membrane contactors for dissolved methane recovery from anaerobic effluents: effect of surface organofluorosilanisation
Source: Environ Sci Pollut Res Int. 2022 Nov 21;30(11):29164–79. doi: 10.1007/s11356-022-24019-z (PMC9995407; doi:10.1007/s11356-022-24019-z)
Supplement: Supplementary file 1 — Supplementary file1 (DOCX 4922 KB) [file 11356_2022_24019_MOESM1_ESM.docx]

**Supplementary Material for**

**Fouling characterization in PVDF membrane contactors for dissolved methane recovery from anaerobic effluents: Effect of surface organofluorosilanisation.**

Ramón Jiménez-Robles, Vicente Martínez-Soria, Marta Izquierdo*

Research Group in Environmental Engineering (GI^2^AM), Department of Chemical Engineering, School of Engineering, University of Valencia, Avda. Universitat s/n, 46100 Burjassot, Spain.

*e-mail: marta.izquierdo-sanchis@uv.es. Phone: +34 354 37 37. Fax: +34 963 54 48 98.

# S1. Characteristics of the anaerobic reactor effluent

Table S 1. Characteristics of the filtered anaerobic reactor effluent. TSS and VSS: total and volatile suspended solids, respectively; COD: chemical oxygen demand, VFA: volatile fatty acids.

| Parameter | **Value** |
| --- | --- |
| pH | 7.84 |
| Conductivity, mS cm^-1^ | 8.61 |
| Turbidity, NTU | 6.7 |
| Alkalinity, mg CaCO_3_ L^-1^ | 2102 |
| TSS, mg L^-1^ | 75.75 |
| VSS, mg L^-1^ | 67.50 |
| COD, mg O_2_ L^-1^ | 414 |
| VFA, mg CH_3_COOH L^-1^ | <5.0^a^ |
| Proteins, mg L^-1^ | 125.06 |
| Polysaccharides, mg L^-1^ | 79.19 |
| NH_4_^+^, mg L^-1^ | 1500 |
| NO_3_^-^, mg L^-1^ | 2500 |
| NO_2_^-^, mg L^-1^ | 400 |
| PO_4_^3-^, mg L^-1^ | 74 |
| SO_4_^2-^, mg L^-1^ | <200^a^ |
| Ca^+^, mg L^-1^ | 50 |
| Fe^2+^, mg L^-1^ | 0 |
| Live cells, L^-1^ | 3.15·10^10^ |
| Dead cells, L^-1^ | 0.40·10^10^ |

*^a^Below detection limit*

The turbidity was measured with a turbidimeter (Merck Turbiquant 1500IR, Germany), volatile fatty acids (VFA) were determined using potentiometer titration (848 Titrino Plus, Metrohm, Switzerland) and a pH and conductivity meter was used (pH/Cond 340i WTW, Germany). The concentration of sulphates (SO_4_^2-^), ammonium (NH_4_^+^), nitrates (NO_3_^-^), nitrites (NO_2_^-^), calcium (Ca^+^) and iron (Fe^2+^) were determined by means of test strips supplier by Merck-Quant.

# S2. Experimental system for the fouling tests

Figure S 1. a) Scheme of the experimental system for the fouling tests with a flat-sheet membrane module using an anaerobic reactor effluent (AE) and b) image of the homemade flat-sheet module used in the tests.

# S3. Images of the evolution of the fouling on the membrane during the fouling tests

Figure S 2. Pictures of the PVDF and modified PVDF (mPVDF) membranes at different operation times of the fouling test with an anaerobic reactor effluent, and after the fouling extraction with a sonication bath in milliQ water and 0.01 M NaOH solution.

# S4. Absorption bands of the infrared spectra of the pristine PVDF membrane

Table S 2. Major FTIR absorption bands obtained in the analysis of the pristine PVDF membrane.

| **Wavenumber, cm^-1^** | **Band** | **Group** | **Reference** |
| --- | --- | --- | --- |
| 762 | PVDF α-crystal shape | PVDF | [1,2] |
| 839 | PVDF β-crystal shape | PVDF | [1,2] |
| 872 | C-F stretching vibration | PVDF | [1,3,4] |
| 975 | PVDF α-crystal shape | PVDF | [1,2] |
| 1070 – 1401 | Fluorocarbon frequencies | PVDF | [4–8] |
| 3024 | C-H (alkenes stretching) | PVDF | [3] |

# S5. Energy Dispersive X-Ray analysis and distribution of different elements on the surface of the PVDF and modified PVDF membranes after the fouling tests

Figure S 3. Distribution of the major elements detected on the surface of the PVDF and modified PVDF (mPVDF) membranes after the fouling tests (operation time > 800 h).

Figure S 4. Distribution of minor inorganic foulants in solid particles detected on the surface of the PVDF and modified PVDF (mPVDF) membranes after the fouling tests (operation time > 800 h). Target particles rounded in red colour.

Figure S 5. Spectra of the EDX analysis over a cluster of particles deposited on the fouling cake of the modified PVDF (operation time > 800 h).

# S6. Energy Dispersive X-Ray analysis and distribution of fluorine on the surface of the PVDF and modified PVDF after the fouling extraction

Figure S 6. Distribution of the fluorine on the surface of the PVDF and modified PVDF (mPVDF) membranes after the fouling extraction. FESEM images of the pristine membrane surfaces are presented in Figure 3 of the main manuscript.

# S7. Energy Dispersive X-Ray analysis and distribution of different elements on the cross section of the PVDF and modified PVDF after the fouling tests

Figure S 7. Distribution of different elements along the cross section of the PVDF and modified PVDF (mPVDF) membranes after the fouling tests (> 800 h). FESEM images of the pristine membrane cross section are presented in Figure 4 of the main manuscript.

# References

[1] H. Dong, K. Xiao, X. Tang, Z. Zhang, J. Dai, R. Long, W. Liao, Preparation and characterization of polyurethane (PU)/polyvinylidene fluoride (PVDF) blending membrane, Desalin. Water Treat. 57 (2016) 3405–3413. https://doi.org/10.1080/19443994.2014.988659.

[2] S. Al-Gharabli, M.O. Mavukkandy, J. Kujawa, S.P. Nunes, H.A. Arafat, Activation of PVDF membranes through facile hydroxylation of the polymeric dope, J. Mater. Res. 32 (2017) 4219–4231. https://doi.org/10.1557/jmr.2017.403.

[3] M.A. Mohamed, J. Jaafar, A.F. Ismail, M.H.D. Othman, M.A. Rahman, Membrane Characterization. Chapter 1: Fourier Transform Infrared (FTIR) Spectroscopy, Elsevier B.V., 2017. https://doi.org/10.1016/B978-0-444-63776-5.00001-2.

[4] Z. Yan, Z. Lu, X. Chen, Y. Jiang, Z. Huang, L. Liu, G. Fan, H. Chang, F. Qu, H. Liang, Membrane distillation treatment of landfill leachate: Characteristics and mechanism of membrane fouling, Sep. Purif. Technol. 289 (2022) 120787. https://doi.org/10.1016/j.seppur.2022.120787.

[5] G.J. Ross, J.F. Watts, M.P. Hill, P. Morrissey, Surface modification of poly(vinylidene fluoride) by alkaline treatment: 1. The degradation mechanism, Polymer (Guildf). 41 (2000) 1685–1696. https://doi.org/10.1016/S0032-3861(99)00343-2.

[6] H. Zhu, X. Li, Y. Pan, G. Liu, H. Wu, M. Jiang, W. Jin, Fluorinated PDMS membrane with anti-biofouling property for in-situ biobutanol recovery from fermentation-pervaporation coupled process, J. Membr. Sci. 609 (2020) 118225. https://doi.org/10.1016/j.memsci.2020.118225.

[7] X. Huang, J. Zhang, W. Wang, Y. Liu, Z. Zhang, L. Li, W. Fan, Effects of PVDF/SiO2 hybrid ultrafiltration membranes by sol-gel method for the concentration of fennel oil in herbal water extract, RSC Adv. 5 (2015) 18258–18266. https://doi.org/10.1039/c4ra15448g.

[8] N. Awanis Hashim, Y. Liu, K. Li, Stability of PVDF hollow fibre membranes in sodium hydroxide aqueous solution, Chem. Eng. Sci. 66 (2011) 1565–1575. https://doi.org/10.1016/j.ces.2010.12.019.
